# What do Turkish, Spanish, and Pakistani medical students value in specialty training positions? A discrete choice experiment

**DOI:** 10.1186/s12909-022-03798-6

**Published:** 2022-11-01

**Authors:** Yavuz Selim Kıyak, Işıl İrem Budakoğlu, Joaquín García-Estañ, Komal Atta, Özlem Coşkun, Emin Koyun

**Affiliations:** 1 Department of Medical Education and Informatics, Gazi University Faculty of Medicine, Ankara, Turkey; 2 Centro de Estudios en Educación Médica, Universidad de Murcia, Murcia, Spain; 3 Department of Medical Education, University Medical and Dental College, The University of Faisalabad, Faisalabad, Pakistan; 4 Department of Cardiology, Sivas Cumhuriyet University, Sivas, Turkey

**Keywords:** Medical students, Discrete choice experiment, Career choices, Specialty training

## Abstract

**Background:**

The aim of this study was to find out specialty training preferences of senior medical students from three medical schools in Turkey, Spain, and Pakistan.

**Methods:**

A Discrete Choice Experiment was carried out using an electronic form for students in three countries in 2021–2022 term. Each choice set in the form consisted of two hypothetical specialty training positions. The attributes were location, earnings, working conditions, personal perspective, quality of education, probability of malpractice, and prestige. Conditional logit model was used to estimate participants’ preferences and “willingness to accept” values.

**Results:**

The most valued attribute was “personal perspective on specialty area” for Turkish and Spanish students, while this attribute was not meaningful for Pakistani students. Turkish students needed a 204% of change in their income for a swap between the specialty that they like and not like. This tradeoff necessitated a 300% change for Spanish students. The most valued attribute for Pakistani students, which was “working conditions”, necessitated a 97% increase in income to switch from working in good conditions to working in poor conditions.

**Conclusion:**

In this first multinational DCE study in the medical education literature, we found the preferences of medical students in Turkey, Spain, and Pakistan are affected to various extents by several factors.

## Introduction

Medical students constitute the future workforce of healthcare institutions. They are the main actors who are going to protect and improve public health in the future. Senior medical students are the closest ones among them in regards to starting work and taking specialty training that they want. There are several factors that affect the senior medical students’ preferences on career choices regards to specialties and subspecialties [1, 2]. Medical school characteristics, student characteristics and values, specialty characteristics, income, workload, status, and prestige are among these factors [1, 2]. These factors have been well-documented in previous studies [1, 2]. However, these studies mostly used descriptive surveys without revealing the relative importance of the factors. Using Discrete Choice Experiment (DCE) to understand their preferences better would fill the gap [3], as it has been utilized to inform health workforce policymakers [4].

DCE is commonly run in market research to understand consumer choices, and to comprehend healthcare workers’ and medical students’ labor preferences to make underserved areas more attractive in developing countries [3, 4]. More recently, DCE was used to understand career preferences of postgraduate students in the United Kingdom (UK) [5–7], and senior medical students in the UK [8] and in China [9]. In these studies, the researchers revealed not only the influential factors on the preferences but also the relative importance of the factors quantitatively with the help of DCE. These were the first studies in terms of using DCE to evaluate the results considering the medical education context. They were, however, limited to the UK and China. Apart from the mentioned studies, a study from the Netherlands [10] has used DCE to determine the preferences of residents on value-based healthcare education. To our knowledge, there is no DCE study that was conducted outside of the UK and China to understand senior medical students’ career preferences.

In order to contribute to filling this gap by revealing the preferences of students in different countries, we aimed to find out the specialty training preferences of senior (sixth-year) medical students from three medical schools which are in Turkey, Spain, and Pakistan. We hypothesized that the preferences of students in different countries would be different, and it would shed light on answering our research question: What are the differences between the preferences of Turkish, Spanish, and Pakistani students on specialty training positions?

## Methods

This quantitative study used DCE to reveal the preferences of medical students in choosing specialty training positions. In DCE studies, participants are asked to choose the best option for them between two or more hypothetical alternatives in every choice set. Alternatives are described by using several attributes and levels. Since participants select a hypothetical training position instead of another, researchers can acquire data about their willingness to trade off these attributes. Therefore, the choices of participants are utilized to determine what factors affect their preferences to what extent [11].

### Development of Choice Sets and Survey Form

DCE User Guide published by World Bank guided us to develop choice sets and survey form [11]. Firstly, we reviewed the existing literature to identify the attributes contributing to medical students’ career choices. We found seven attributes with different levels. The attributes and their levels are presented in Table 1.

**Table 1:** Descriptions and Levels of the Attributes of Specialty Training Options for Senior Year Medical Students

Attribute	Description	Levels
Location	It refers to the geographical location of the training position, including the amenities on offer and the proximity to your family/friends.	The Location You Do Not Desire The Location You Desire
Earnings	It refers to how your potential earnings compare against average career earnings in specialties after completing training.	Average 20% Above the Average 40% Above the Average
Working Conditions	It refers to conditions, such as shift hours, amount of on-call, time off, etc.	Poor Moderate Excellent
Personal Perspective on Specialty Area	It refers to whether you like or do not like the specialty area.	Not Like Moderately Like Really Like
Quality of Education	It refers to opportunities to improve yourself in your specialty area to be more competent.	Low High
Probability of Malpractice/Being Sued	It refers to whether the specialty is more prone to commit malpractice or being sued by patients.	Low High
Prestige/Reputation in the Eyes of Community	It refers to how the public, family or friends see the specialty area. How prestigious is it? Does it have high reputation?	Low High

Each choice set consisted of two hypothetical specialty training positions. We generated choice sets by using the choice design facility of JMP Statistical Software 13 (SAS Institute Inc, Cary, NC, USA) to ensure an effective design of the sets. The software program generated 13 pairs of specialty training positions.

The first part of the survey form included questions that ask the demographic characteristics of the participants (sex and age), and whether students want to take the specialty training exam. These specialty exams are “TUS” (Tıpta Uzmanlık Sınavı) for Turkey, “MIR” (Médico Interno Residente) for Spain, “FCPS” (Fellowship of the College of Physicians and Surgeons) for Pakistan. Specialty training positions were in the second part as well as explanations of the hypothetical alternatives and levels. The participants were obliged to choose one position that fits better for them among two alternatives. There was no opt-out option. Instructions and one of the choice sets are presented in Table 2.

The survey form was developed in English. Subsequently, it has been translated into Spanish and Turkish by Spanish and Turkish native speakers. In order to be sure that there is no mistranslation, the forms were retranslated by volunteer translators into English. Moreover, one medical student (who speaks English) checked for each language if the translated form reflects the original form and is clear. Since the medium of teaching in the Pakistani medical school is English, we applied the survey in English.

**Table 2:** Instructions for Participants and Choice Set No.1

In this part of the questionnaire, you are going to read pairs of specialty training options. Every question consists of two different hypothetical specialty training options that differ in characteristics in seven titles. Imagine you will start a specialty education and you have two alternatives. These options only differ in the characteristics outlined below. All other unmentioned characteristics are the same. • “Location” refers to the geographical location of the training position, including the amenities on offer and the proximity to your family/friends. • “Earnings” refers to how your potential earnings compare against average career earnings in specialties after completing training. • “Working Conditions” refers to conditions, such as shift hours, amount of on-call, time off, etc. • “Perspective on Specialty Area” refers to whether you like or do not like the specialty area. • “Quality of Education” refers to opportunities to improve yourself in your specialty area to be more competent. • “Probability of Malpractice/Being Sued” refers to whether the specialty is more prone to commit malpractice or being sued by patients. • “Prestige/Reputation in the Eyes of Community” refers to how the public, family or friends see the specialty area. How prestigious is it? Does it have high reputation? You may not like either position but we would like you to choose which is better in your opinion! Don’t forget, there is no true or false answer. All we need is to understand your personal perspective.		
**Choice Set No.1**		
**Parameters**	**Option A**	**Option B**
Location	The Location You Desire	The Location You Do Not Desire
Income	40% Above the Average	20% Above the Average
Working Conditions	Excellent	Poor
Personal Perspective on Specialty Area	You Moderately Like	You Really Like
Quality of Education	Low	High
Probability of Malpractice/Being Sued	High	Low
Prestige/Reputation in the Eyes of Community	Low	High
Which One Do You Prefer?	A ☐	B ☐

### Participants and data collection

Senior medical students from a medical school in Turkey (Gazi University/GU), one in Spain (Universidad de Murcia/UM), and one in Pakistan (The University of Faisalabad/TUF) participated in the study. While GU and UM were public universities, TUF was a private university. The selection of the faculties was only based on convenience. The total number of senior medical students in each medical faculty was, GU: 349, UM: 200, TUF: 145. We did not carry out any formal sample size calculation since more than 30 participants are sufficient in DCE to conduct meaningful statistical analysis [11]. The survey forms were sent to students in an electronic environment (Google Forms) in 2021–2022. In all the participant medical faculties, the students were in face-to-face practices during the data collection period.

### Preference analysis

The assumption of our study was that the participants have chosen the training position that would provide the highest utility from the alternatives in every choice set. Based on this assumption, conditional logit model [11] was used to estimate participants’ preferences. All of the attributes were qualitative and entered into the data sheets as dummy-coded variables.

Statistical analysis was conducted using Stata 13 (StataCorp LP, College Station, TX, USA).

We assumed that the utility is linear and additive, as the following function of the attributes:

V = ß_0_ + ß_1_location_desired + ß_2_income_40above + ß_3_income_20above + ß_4_workingconditions_excellent + ß_5_workingconditions_moderate + ß_6_area_reallylike + ß_7_area_moderatelylike + ß_8_quality_high + ß_9_malpractice_low + ß_10_prestige_high + ε_i_.

“V” refers to the utility derived from a given training position. “ε_i_“ shows the error term.

Willingness to accept (WTA) analysis provided a calculation to see what the monetary equivalent of a change is, for example, from a good working condition to the bad one. In this study, we reported WTA values as the necessary percentage for a certain change (e.g. “-50” means that I accept a 50% decrease in my salary to work in a desirable location instead of an undesirable one). We calculated WTA values and their confidence intervals (CI) using the delta approach in Stata as explained in DCE User Guide [11]. The coefficients were accepted as statistically significant if their p-values are below 0.05 (5% level).

Although the analysis was carried out using conditional logit model, we repeated the analysis using mixed logit model in Stata in order to reveal the evidence about the validity of DCE. The results were not substantially different between these models. It supports that the use of model specifications is appropriate. Therefore, it is a sign that strengthens the theoretical validity of our DCE implementation.

### Ethical considerations

The survey form did not include any detail that can identify the participants. It was blocked to access the survey form without approving informed consent. Gazi University Institutional Review Board has approved the study (code: 2020 − 482).

## Results

The number of students who responded to the survey was 211 (60.4%) in Turkey, 170 (85.0%) in Spain, and 110 (75.8%) in Pakistan. There was no missing response. Descriptive statistics about the participants’ sex, age, and attendance to specialty exams are presented in Table 3.

**Table 3:** Descriptive statistics

	Number of Respondents		
	**Turkey (%)**	**Spain (%)**	**Pakistan (%)**
Sex			
Female	105 (49.7)	119 (70.0)	74 (67.3)
Male	106 (50.3)	51 (30.0)	36 (32.7)
Age (years)			
18–23	80 (37.9)	142 (83.5)	62 (56.4)
24–29	125 (59.3)	15 (8.8)	47(42.7)
30 or above	6 (2.8)	13 (7.7)	1 (0.9)
Are you planning to attend TUS/MIR/FCPS exam?			
Yes	196 (92.9)	170 (100.0)	95 (86.4)
No	15 (7.1)	0 (0)	15 (13.6)

According to the coefficients extracted from conditional logistic regression, out of nine levels, the results were statistically meaningful for Turkish students in eight levels, for Spanish students in seven levels, and for Pakistani students in only five levels. “Prestige” was not meaningful for Spanish and Pakistani students. Moreover, “personal perspective on specialty area” and “probability of malpractice/being sued” were not found meaningful in Pakistani students’ preferences. However, these two attributes were meaningful for both Turkish and Spanish students.

The most valued attribute was “personal perspective on specialty area” for Turkish (CE/coefficient: 1.18) and Spanish (CE: 2.74) students, while this attribute was not meaningful for Pakistani students (p > 0.05). Turkish students needed a 204% of change in their income for a swap between the specialty that they like and not like. This tradeoff necessitated a 300% change for Spanish students. The most valued attribute for Pakistani students, which is “working conditions”, necessitated a 97% increase in income to switch from working in good conditions to working in poor conditions. “Quality of education” was among the top valued attributes for all three of the countries. Table 4 presents all results.

**Table 4:** Results of conditional logistic regression and willingness to accept (WTA) analysis

	Turkey (GU)		Spain (UM)		Pakistan (TUF)	
Attribute (Model Parameters)	Coefficient (SE)	WTA (CI)	Coefficient (SE)	WTA (CI)	Coefficient (SE)	WTA (CI)
Income	0.005* (0.002)		0.009* (0.003)		0.007* (0.002)	
Location (relative to undesirable city)						
Desirable	0.35* (0.13)	-61.09 (-129.56; 7.37)	0.88* (0.19)	-96.73 (-191.99; -1.47)	0.21* (0.06)	-30.71 (-56.58; -4.83)
Working Conditions (relative to poor conditions)						
Neutral	0.25 (0.12)	-43.60 (-92.79; 5.58)	0.15 (0.17)	-17.21 (-53.99; 19.56)	0.15* (0.07)	-22.73 (-48.04; 2.57)
Good	1.01* (0.21)	-174.30 (-310.02; -38.58)	0.77* (0.32)	-84.85 (-159.44; -10.25)	0.38* (0.08)	-54.96 (-97.62; -12.30)
Personal Perspective on Specialty Area (relative to not liked area)						
Neutral	0.68* (0.06)	-117.48 (-209.95; -25.00)	1.37* (0.09)	-150.00 (-265.27 -34.74)	0.08 (0.07)	-12.58 (-35.79; 10.61)
Liked	1.18* (0.08)	-204.14 (-364.61; -43.66)	2.74* (0.13)	-300.71 (-534.77; -66.65)	0.06 (0.08)	-8.72 (-34.68; 17.22)
Quality of Education (relative to low quality)						
High	1.16* (0.16)	-201.21 (-353.94; -48.48)	0.82* (0.21)	-90.01 (-163.94; -16.08)	0.26* (0.07)	-37.44 (-70.93; -3.95)
Probability of Malpractice/Being Sued (relative to high probability)						
Low	0.50* (0.18)	-87.11 (-184.71; 10.47)	0.79* (0.26)	-86.57 (-182.45; 9.31)	0.07 (0.07)	-11.02 (-32.05; 10.00)
Prestige/Reputation in the Eyes of Community (relative to low prestige)						
High	0.28* (0.07)	-49.42 (-97.05; -1.78)	0.19 (0.12)	-21.66 (-55.93; 12.60)	0.11 (0.07)	-16.50 (-55.93; 12.60)
Statistics						
Participants	211		170		110	
Observations	5486		4420		2860	
Parameters	13		13		13	
Log-likelihood	-1464.897		-864.835		-970.5498	

*p < 0.05, SE: Standard Error, CI: Confidence Interval, GU: Gazi University, UM: Universidad de Murcia, TUF: The University of Faisalabad

## Discussion

To our knowledge, this is the first multinational study that utilizes DCE to reveal the relative importance of senior medical students’ preferences in specialty training positions. We found that students in different countries have different preferences as previous studies showed in the UK [8] and China [9].

In our study, “personal perspective on specialty area” was the most valued attribute in Turkey and Spain. It implied that Turkish and Spanish students are determined to chase after the specialty areas that they really like. However, the most valued attribute was “working conditions” in Pakistan, similar to the study conducted in the UK [8]. Moreover, almost all coefficients of the attributes were statistically significant in Turkish and Spanish students both, whilst only a few were significant in Pakistani students. Although Turkey, Spain, and Pakistan have very similar routes from the beginning of medical school to becoming a specialist [12], these results showed one more time that the community of practice [13] in each country has different characteristics. Their preferences during legitimate peripheral participation [14] may be affected by their unique contextual factors. As evidence for this point of view, a recent systematic review showed that type of medical school, ethnicity, and geography in which students live are among the factors associated with medical career preferences [1]. Our study also showed that these differences make the preparation of a multinational DCE more difficult because it is quite impractical to find attributes that are meaningful in all of the countries included in a study. Statistically non-significant attributes found in Pakistan are a solid sign of that.

Apart from the differences, the preferences of the students in all three countries were significantly affected by non-monetary attributes as well in each country to various extents, which is consistent with previous studies [8, 9]. These results, especially with WTA analysis, provide important opportunities for healthcare policymakers to find ways to create tailored solutions regarding the preferences of the students in each country. For instance, without changing salaries, only turning “poor” working conditions into “good” can serve as if the salaries are increased by around 310% in Turkey, 159% in Spain, and 97% in Pakistan. From another point of view, policymakers can be aware of what they should not focus on as well. For example, by considering that it necessitates a 534% increase in their salaries, policymakers can notice the difficulty to get Spanish students to choose a specialty that they do not like. In this way, policymakers could choose more efficient strategies for determining incentives.

The specialization trends from 1987 to 2017 showed that it could create serious problems for sustainable healthcare services in Turkey because some specialties are strongly demanded by students while some specialties cannot fill even half of their available positions [15, 16]. One of the prominent reasons stated by Turkish students avoiding certain specialties is, similar to our results, poor working conditions in these specialties [17]. Similarly, the specialization trend is a serious problem in Spain, especially for primary care [18]. Due to several reasons including this trend, there are some attempts to change the structure of the specialty exam (MIR) in Spain [19]. Besides, the lack of structured supervisorship, dwindling geopolitical conditions, and poor living and working conditions in Pakistan factor in this study [20]. These studies show that policymakers and educational innovators in these countries will lead to critical changes. Before embarking on the changes, they are going to need data to support “conceptual foundation of innovation” [21]. The results of our study could be one of valuable sources for them to solve these problems and make changes accordingly to the actual conditions.

Even if this study is the first multinational DCE to reveal the career preferences of medical students, it has limitations. The prominent one is that it samples only one medical school for each country. Therefore, the results cannot be generalizable across these countries. Future research can reach more medical schools instead of only one in each country. Another limitation is that the preference heterogeneity analysis could not be carried out due to various reasons. One important limitation originated from the nature of DCE since it is one of the stated preference methods [22] which do not show the actual preferences in real life. Even if we have provided evidence about the theoretical validity of our DCE implementation, we could not show evidence for external validity. It would be beneficial to follow the students after graduation to see if their actual choice will be aligned with their choices in the DCE. Another limitation is that the survey was applied in different languages. The differences in the composition of the population of each school, the percentages of responses in terms of females and males, and the school types (GU and UM were public, TUF was private) are among the limitations of this study.

## Conclusion

In this first multinational DCE study in the medical education literature, we found that the preferences of medical students in Turkey, Spain, and Pakistan are affected to various extents by several factors including non-monetary ones. The results of our study hopefully will shed light on the ways of the policymakers and educational innovators in these countries that are on brink of the changes regarding the medical specialization process.

## Data Availability

The datasets generated and/or analysed during the current study are not publicly available due to institutional regulations but are available from the corresponding author on reasonable request.
